# Prognostic Role of Neutrophil-to-Lymphocyte Ratio in Patients with COVID-19

**DOI:** 10.3390/jcm11164688

**Published:** 2022-08-11

**Authors:** Daniele Piovani, Andreas G. Tsantes, Stefanos Bonovas

**Affiliations:** 1 Department of Biomedical Sciences, Humanitas University, Pieve Emanuele, 20090 Milan, Italy; 2 IRCCS Humanitas Research Hospital, Rozzano, 20089 Milan, Italy; 3 Laboratory of Haematology and Blood Bank Unit, “Attiko” University Hospital, School of Medicine, National and Kapodistrian University of Athens, 12462 Athens, Greece; 4 Microbiology Department, “Saint Savvas” Oncology Hospital, 11522 Athens, Greece

The coronavirus disease 2019 (COVID-19) pandemic, caused by the novel severe acute respiratory syndrome coronavirus 2 (SARS-CoV-2), has had a major impact on global health, continuing to put strain on healthcare systems and disrupting socioeconomic life. Since the start of the pandemic in late 2019, SARS-CoV-2 has taken more than six million human lives (as of 31 July 2022) and continues to spread worldwide with more than 570 million confirmed cases [1].

Due to COVID-19 displaying a very heterogeneous clinical behavior (i.e., the majority of patients are asymptomatic or suffer mild symptoms, while others demonstrate an aggressive and life-threatening disease), the early detection of high-risk patients is crucial. Several research studies have sought to identify markers correlating with disease severity and prognosis, while requiring routine and widely available diagnostic/laboratory testing [2]. As a growing body of evidence suggests that inflammatory mechanisms play a pivotal role in COVID-19-related organ dysfunction and mortality [3,4], the potential role of the neutrophil-to-lymphocyte ratio (NLR) in COVID-19 prognosis has been extensively investigated.

The NLR is a cheap and easy-to-obtain biomarker, calculated as the ratio between the neutrophil and lymphocyte counts measured in peripheral blood. It mirrors the balance between two aspects of the immune process, i.e., (i) the innate immune response, mainly due to neutrophils, and (ii) adaptive immunity, supported by lymphocytes [5]. Well before the COVID-19 pandemic, it was recognized that an elevated NLR has been observed in conditions characterized by tissue damage that activates the systemic inflammatory response, such as bacterial and fungal infections, sepsis, acute stroke, atherosclerosis, myocardial infarction, severe trauma and cancer [6]. A systematic review of diagnostic and prognostic factors for COVID-19 patients, published in the *BMJ*, identified that lymphocyte and neutrophil counts were frequently included in published multivariable diagnostic and prognostic models [7].

Since the start of the pandemic, a series of articles in the *Journal of Clinical Medicine* shed light on the prognostic role of the NLR in COVID-19. As discussed below, the NLR was shown to predict mortality, progression to severe disease and admission to the intensive care unit (ICU), capable of risk-stratifying COVID-19 patients and help medical decision making (i.e., accurate triage and treatment).

In 2021, a multicenter retrospective cohort study from France [8] investigated the prognostic value of the NLR for disease severity and mortality in 1035 COVID-19 patients. The NLR at admission to the emergency department had a capacity to predict disease severity (area under the curve (AUC), 0.59; cut-off value, 6.88; sensitivity, 48%; specificity, 66%) and disease mortality (AUC, 0.62; cut-off value, 8.23; sensitivity, 47%; specificity, 72%). Similarly, a large case–control study from the USA [9] analyzed 4103 COVID-19 patients and found that an elevated NLR (cut-off value, 5.1) was an early predictor of poor outcomes (mechanical ventilation, death or discharge to hospice). Another large retrospective cohort study from the USA [10] searched for factors associated with “care transitions” (i.e., transition from the emergency department to the inpatient floor and also to the ICU) by analyzing data from 11,406 COVID-19 patients. Among laboratory variables, the NLR was associated with transitions to higher levels of care.

In 2022, a single-center retrospective cohort study from Greece [11], conducted on a selected population of 241 unvaccinated patients without comorbidities, confirmed that an elevated NLR predicted the development of critical disease (AUC, 0.77). A two-center retrospective cohort study from Italy [12] analyzed 411 hospitalized COVID-19 patients and showed that the NLR could predict in-hospital mortality (AUC, 0.77; cut-off value, 11.38; sensitivity, 73%; specificity, 72%) as well as disease progression to a stage of severity that required an ICU admission (AUC, 0.66; cut-off value, 8.21; sensitivity, 81%; specificity, 49%). A retrospective cohort study from Israel [13], conducted on 762 patients, demonstrated that an elevated NLR predicted in-hospital mortality (AUC, 0.68) and was the prognostic marker of choice, particularly for older patients (66–80 years) with COVID-19.

Regarding the prognosis of COVID-19 patients admitted to the ICU, a small retrospective cohort study from Poland [14] included 70 subjects with acute respiratory failure, and demonstrated that the NLR on admission to the ICU could accurately predict patient death (AUC, 0.76; cut-off value, 14.38; sensitivity, 77%; specificity, 69%). Similarly, a two-center retrospective cohort study from Romania [15], conducted on 425 critically ill patients requiring ICU admission, confirmed that the NLR, together with age and the SOFA score, were independent predictors of mortality.

To sum up, the NLR has emerged as a predictor for the evolution and prognosis of COVID-19, which is of high clinical interest, particularly considering its rapid, widely available and relatively cheap assessment through routine blood count analyses. The current evidence suggests a clear association between elevated NLR values and the occurrence of poor outcomes in COVID-19. Moreover, SARS-CoV-2 continues to evolve, and several variants have emerged in the last two years. In this ever-changing landscape, continuing to study the NLR and other factors that aid patient stratification remains crucial. Large, well-designed, multicenter prospective cohort studies in patients with various disease phases and different therapies are needed to better define the clinical role of the NLR in COVID-19.
